# Editorial: Collaborative interaction and control for intelligent human-robot systems

**DOI:** 10.3389/fnbot.2023.1147663

**Published:** 2023-02-13

**Authors:** Rui Huang, Kecheng Shi, Xin Li, Kaibo Shi

**Affiliations:** ^1^School of Automation Engineering, University of Electronic Science and Technology of China, Chengdu, China; ^2^Group 42, Abu Dhabi, United Arab Emirates; ^3^School of Information Science and Engineering, Chengdu University, Chengdu, China

**Keywords:** human-robot collaboration (HRC), human-robot interaction (HRI), intelligent control, human-robot systems, motor learning

Human-Robot Collaboration (HRC) studies collaborative processes in which humans and robots work together to achieve shared goals. Humans and robots form intelligent human-robot systems and cooperate, which could achieve better performances in terms of safety and efficiency in cooperative tasks. This special issue is dedicated to researching collaborative interaction and control technologies for intelligent human-robot systems. It is focused on the bi-directional human-robot interaction (HRI) and motor collaborative control methods in behavior-based cooperative tasks. Seven articles are received by this special issue, and the contents of these articles are briefly described as follows.

The lower limb exoskeleton is playing an increasing role in assisting patients with spinal cord injury with some daily movements. However, the balance of the human-exoskeleton system is challenging to maintain. It is essential to maintain the balance of the human-exoskeleton system during movements. Xu et al. have proposed a novel Enhanced Stability Pyramid Index (ESPI) and Dynamic Movement Primitives (DMPs)-based balance control strategy for the human-exoskeleton system during movements. ESPI uses eXtrapolated Center of Mass to assess the safety of human-exoskeleton systems, and combines the DMPs method to generate the human-robot gait trajectory. Finally, the walking simulation in Gazebo and the experiments of the human-exoskeleton system have verified the effectiveness of the balance control strategy.

In unknown interaction environments, the ability to quickly learn a new skill is the key point in the HRC field. It usually is helpful to have an expert guiding through unknown environmental dynamics. Saracbasi et al. have tested the effectiveness of three strategies for skill transfer: practice alone, together with another beginner, and learning from the expert in a cooperative visual-haptic motor task, and evaluated the adaptability of the novice participants to a new partner while attempting to achieve a common goal together. The experiment results have shown that peer-to-peer interactions among paired beginners enhanced the motor learning most, and individuals practicing on their own (learning as a single) showed better motor learning than practicing under the expert's guidance.

Compared to robots, humans have the amazing capability to handle complex and highly uncertain tasks. Transferring human manipulation skills to robots can significantly improve their ability to handle complex tasks, and adapt to the flexibility of the manufacturing system. Si et al. have designed an impedance-based control architecture of telemanipulation in task space for the human-robot skill transfer through teleoperation. This framework achieves human-robot skill transfer and provides a solution to human-robot collaboration through teleoperation. The performance of the proposed approach was evaluated on a 7-DoF Franka Panda through the robot-assisted composite layup on different shapes and orientations of the components. Results have shown that the tracking error of our approach is <0.005 m, which is feasible for the composite layup.

The signals from electromyography (EMG) have been used for volitional control of robotic assistive devices. Recently have made some good progress in the HRI field. The performance accuracy of EMG-based robot control systems is often affected by noise or artifacts and lacks robustness for various subjects due to individual biological variability. Chen B. et al. have developed an EMG-based fixed-bandwidth frequency-domain embedded system for volitional robot movement control. The system recruited healthy volunteers to identify the optimal myoelectric signal frequency bandwidth of muscle contractions, which could achieve an average motion recognition accuracy of 91.55% with a motion recognition time delay of 300 ms.

An effective control of exoskeleton robots using the human-robot interface is crucial for assessing the robot's movements and the force they produce to generate effective control signals. However, the review papers that were previously published have not thoroughly examined the control strategy which is a crucial component of automating the exoskeleton systems. Masengo et al. have examined the most recent developments and problems associated with exoskeleton control systems. In addition, the trends and challenges of the cooperative control, particularly the multi-information fusion was discussed.

Human-robot interfaces based on surface electromyography (sEMG) have been widely used in lower-limb exoskeleton robots. However, accurate and efficient lower-limb movement prediction remains a challenge due to complex movement information and individual differences. Yang et al. have proposed a human-exoskeleton interface for patients with hemiplegia. The interface framework (HCSNet) was constructed by fusing time and frequency domain hand-crafted features and channel synergy learning-based features. Experimental results have shown that the interface can achieve 95.93 and 90.37% prediction accuracy in both within-subject and cross-subject cases.

Collaborative state recognition is critical for physical human–robot collaboration (PHRC). Chen S. et al. have proposed a contact dynamics-based state recognition method to identify the human–robot collaborative grinding state. This method established two contact dynamic models to identify the difference in dynamics between the human–robot contact and the robot–environment contact. Then, Spearman's correlation and random forest recursive feature elimination were used to feature selections. Long short-term memory was used to construct a collaborative state classifier. Experimental results have illustrated that the proposed method can achieve a recognition accuracy of 97% in a period of 5 ms and 99% in a period of 40 ms.

## Author contributions

RH, XL, and KaS discussed the structure of this manuscript and revised this manuscript for three times. KeS initialized the manuscript. All authors contributed to the article and approved the submitted version.

